# Exploring the Relationship Between Socio-Demographic Factors and Consumers’ Perception of Food Promotions in Romania

**DOI:** 10.3390/foods14040599

**Published:** 2025-02-11

**Authors:** Nicoleta Defta, Andreea Barbu, Violeta Alexandra Ion, Elena Narcisa Pogurschi, Aurelia Osman, Liviu-Cristian Cune, Liliana Aurelia Bădulescu

**Affiliations:** 1 Faculty of Animal Productions Engineering and Management, University of Agronomic Sciences and Veterinary Medicine of Bucharest, 59 Marasti Blvd., 011464 Bucharest, Romania; nicoleta.defta@usamv.ro (N.D.); elena.pogurschi@usamv.ro (E.N.P.); auraosman@gmail.com (A.O.); 2 Research Center for Studies of Food Quality and Agricultural Products, University of Agronomic Sciences and Veterinary Medicine of Bucharest, 59 Marasti Blvd., 011464 Bucharest, Romania; andreea.stan@qlab.usamv.ro (A.B.); liliana.badulescu@qlab.usamv.ro (L.A.B.); 3 Department of Theoretical Physics, Horia Hulubei National Institute of Physics and Nuclear Engineering, 077125 Măgurele, Romania; cune@theory.nipne.ro; 4 Faculty of Horticulture, University of Agronomic Sciences and Veterinary Medicine of Bucharest, 59 Marasti Blvd., 011464 Bucharest, Romania

**Keywords:** respondent profile, price discounts, surveys, marketing mix, consumer behavior, advertising, price promotions

## Abstract

In the context of economic crises and inflationary pressures, consumers often rely on discounted food products to manage their monthly budgets. This study aims to explore how socio-demographic factors are associated with Romanian consumers’ perception of food promotions. Data were collected from 1060 respondents, and the analysis was conducted using R version 4.4.2, applying both descriptive and inferential statistical methods, including Pearson’s chi-square test and multinomial logistic regression. The Pearson’s chi-square test revealed significant differences in consumer responses based on all socio-demographic factors examined, except for residence. Males, married individuals, those with higher education, and higher net incomes were generally more cautious about promotions, while younger consumers (aged 18–24) showed greater receptiveness. The multinomial logistic regression further identified significant predictors and estimated their impact on consumers’ perception of food promotions. We found that *gender*, *marital status*, *education level*, and *age* were strong predictors, while *income* had only a quadratic impact, and *residence* showed no statistically significant outcome. These findings offer valuable insights for shaping marketing strategies and highlight the role of socio-demographic factors in shaping consumer perceptions toward food promotions.

## 1. Introduction

Promotional strategies are commonly employed by retailers to boost sales by fostering consumer loyalty and attracting new customers. The primary objectives of discounts and exclusive offers are to encourage customers to try new brands, purchase in bulk, and increase their overall spending. Sales promotions play a significant role in influencing shoppers’ final purchasing decisions [1]. For food products, the main types of promotions include price discounts (e.g., 50% off, coupons, loyalty card discounts, refunds), extra-product price promotions (e.g., “buy one, get one free”, discounts on a second item, multi-purchase deals, bonus-size packages), premium promotions, collector promotions, prize promotions, and sampling promotions [2].

The environment in which food price promotions are displayed—such as at the front of the store, at the end of the aisle, in the aisle, on shelf talkers, on in-store flyers or banners, on food packaging, or on the first page of a store’s website—also significantly influences consumers’ purchasing behavior both online and offline [2]. On social networks, promotions serve as primary marketing tools, helping increase sales across both online and offline platforms [3].

Price reductions for food products in offline environments are typically available to all customers [4] and often have a more significant impact, particularly on loyal consumers of the promoted product [5]. For instance, a study examining purchasing behavior for orange juice found that price reductions had a notable effect on offline purchases.

The decision-making process behind purchasing food products is complex and influenced by various factors, including marketing strategies, the psychology of promotions [6], the pyramid of purchasing needs (which encompasses food/survival, the need for security, the need for belonging and affection, the need for recognition, and the need for achievement/status) [7], the impact of digital influences [8], and socio-economic influences [9,10].

Purchase decisions are significantly influenced by consumer preferences, perceptions, and behaviors, all of which are shaped by multiple factors [11]. These factors can be intrinsic (e.g., consumer preference for color, taste, and texture) or extrinsic (e.g., packaging, brand, and origin), and are further influenced by socio-economic and demographic characteristics [12,13]. In many studies, socio-economic and demographic factors have been used to explain consumer attitudes and purchasing behaviors [14]. However, the findings of these studies have been mixed. While some researchers have found that socio-economic and demographic factors play a significant role in shaping consumer behavior, others have reported contrasting results [12,13,14].

Over the years, researchers from diverse fields such as psychology, anthropology, economics, and sociology have conducted studies and developed theories to better understand consumer purchase motivations and behaviors [15]. Among these, the Theory of Planned Behavior (TPB) has proven effective in explaining and predicting consumer behaviors [10,16,17,18,19]. While this study does not directly apply TPB as a theoretical framework, its core principles—such as the role of attitudes, subjective norms, and perceived behavioral control—are relevant to understand factors that influence consumer purchasing behavior. Since purchase intent serves as an intermediate step and a strong predictor of actual buying behavior, gaining insights into these influencing factors can help businesses tailor marketing strategies that better meet consumer needs and drive sales.

Emphasizing the positive psychological effects of obtaining a “bargain” highlights its influence on the perception of product value. For instance, the satisfaction a consumer experiences when using a purchased product can be seen as an immediate reward. From this perspective, price promotions not only boost sales, but also enhance consumer enjoyment when using the product, thereby fostering loyalty [20,21].

Understanding socio-demographic factors (e.g., gender—male or female; residence—urban or rural; age groups; educational levels) associated with the intention to purchase certain food products allows for the customization of promotions for these specific groups. Furthermore, different socio-demographic categories have varied reasons for purchasing food products, leading to distinct consumption patterns [22,23]. A study by Zhao et al. (2021) [24] explored how prices and product information influence consumer behavior, highlighting the significance of temporary discounts and their impact on buyer decision-making.

To better understand how socio-demographic factors shape consumer behavior, it is essential to consider how specific characteristics, such as gender, influence purchasing decisions. Men and women have different perceptions, considerations, and cognitive abilities, making gender an important factor in food purchasing [25]. For instance, one study found that women are primarily responsible for purchasing household food [26] and tend to be more careful with their predetermined budgets. This heightened sensitivity to price changes and promotional offers may influence their purchasing behavior [6,7]. Additionally, Xu et al. (2024) [27] observed that modern women are no longer satisfied with traditional product selection; their purchasing preferences and behavior are now influenced by factors such as economic independence, personal values, and shifting cultural norms. Therefore, marketing professionals must gain a deeper understanding of women’s consumption behavior [27] to develop strategies and promotions that will effectively capture their attention.

In addition to gender, age is another crucial socio-demographic factor that significantly influences consumer behavior. Raj et al. (2020) [25] observed that consumer behavior changes with age, with younger individuals tending to prefer convenience, fun, and tasty foods, while older individuals are more focused on foods with health benefits. Young consumers are often influenced by social interactions and trends, being more likely to purchase popular or endorsed products within their social circles. When promotions align with these social trends, young consumers may be more inclined to buy foods that are not necessarily healthy [28,29]. Promotions offer young people the opportunity to purchase desired products without exceeding their budget and provide access to high-quality, branded products that are often associated with a certain social status.

Another important factor influencing purchasing decisions is marital status, followed by the number of family members and the presence of children under the age of 6 years in the household [25]. Additionally, individuals with higher education tend to spend more time researching and purchasing food products. They demonstrate a greater awareness of marketing strategies and are less influenced by promotional pricing. These consumers prioritize quality and safety over discounts [30,31]. Furthermore, they are more likely to favor organic products, which are rarely promoted, and emphasize sustainability and quality rather than promotional deals [25,30,31].

Krige et al. (2012) [32] found that socio-demographic factors such as ethnicity, age, and gender are strongly associated with food safety concerns, particularly among Europeans with a lower socio-economic status (SES) who tend to eat less healthily than those with a higher SES. In this context, individuals with a lower SES are more likely to accept food promotions. People with lower incomes often rely on quick, intuitive decisions and view promotions as valuable opportunities for savings [33,34]. In contrast, higher-income individuals, who are accustomed to specific brands and high-quality products, may still purchase discounted items from the same range, even though they can afford to buy full-priced alternatives [35,36].

Another factor influencing consumer behavior is residence, with rural and urban areas exhibiting distinct characteristics. In rural areas, factors such as poor infrastructure, large family size, and low income significantly shape consumer behavior. In contrast, urban consumers are influenced by the strong presence of organized retail, smaller family sizes [37], and higher income levels [38]. The unequal distribution of income is one of the key differences between rural and urban consumers [37]. As a result, rural consumers are more cautious in their purchasing decisions and often require incentives such as free samples, the ability to touch products, smaller package sizes, and price promotions to encourage them to try new brands [37]. Additionally, the rise of online shopping and home delivery services has made online promotions and delivery options increasingly attractive to consumers in both urban and rural areas.

This dynamic interaction between socio-demographic factors and purchasing behavior also extends to the way consumers perceive and respond to promotional strategies. Just as marketers employ strategic promotional tactics, consumers also develop their own strategies to maximize personal economic benefits. These consumers, referred to as “strategic consumers”, carefully evaluate conditions such as waiting for promotions, discounts, or special offers before making a purchase [39,40,41,42]. Research has shown that socio-demographic factors, such as gender, age, education level, and income, influence how consumers react to promotions, the timing of their purchases, and their overall purchasing decisions [43].

In line with this, understanding how consumers balance their economic and psychological perceptions of promotions is crucial for effectively designing promotional strategies.

Among the factors influencing purchase decisions, we can identify one key element: the general perception of promotions. This involves the consumer’s evaluation of perceived quality and confidence in the legitimacy of the offers. Purchase intention is directly influenced by these perceptions, which, in turn, significantly affect the purchase decision. The process of interpreting promotional messages is not passive; consumers actively engage with these messages to determine their value and relevance. By capturing attention and offering positive interpretations, promotions can directly influence purchasing behavior. Therefore, measuring perception (e.g., through a Likert scale) allows for a deeper understanding of consumers’ relationships with promotions, providing valuable insights for developing targeted marketing strategies.

Building on this, the present study explores whether and how socio-demographic factors are associated with consumers’ perception of food promotions, aiming to bridge existing gaps in research. As discussed earlier, the direct link between socio-demographic factors and purchase intention and/or decision has often been addressed. In this study, we focus on how these factors influence the initial stage of this process, particularly consumer perceptions of food promotions. Furthermore, this research offers new insights into consumer behavior in Romania, particularly in the context of current economic and financial dynamics. Statistical differences in consumer perceptions of promotions across socio-demographic factors are assessed using the Pearson’s chi-square test, and socio-demographic predictors of consumer perceptions are analyzed through a multinomial logistic regression model.

## 2. Materials and Methods

### 2.1. Participants, Recruitment, and Procedure

This study analyzes the socio-demographic factors related to consumers’ perceptions of food promotions in Romania using a cross-sectional observational questionnaire [44,45,46,47]. The questionnaire was distributed through online platforms from October 2021 to June 2022. It was available to all who accessed the online platforms without any restrictions or rewards.

The questionnaire was structured into two sections. The first section focused on socio-demographic factors, collecting data on participants’ gender, area of residence (urban or rural), education level, income, and marital status. The second section aimed to profile the consumer, including questions about the health status, frequency of consumption of dried vegetables or products made from dried vegetables, preferences for organic versus traditional chips, and consumer perceptions of food promotions. One of the key items in the study was: “*To what extent do promotions influence food choices?*”. Respondents were asked to select their answer using the following Likert scale: strongly disagree, disagree, neutral, agree, and strongly agree. The use of a Likert scale allowed for the quantification of factors underlying consumers’ perceptions of food promotions.

A total of 1060 participants, comprising both male and female individuals from a variety of socio-demographic backgrounds, completed the questionnaire. Participants were informed about the purpose of the research and were given the option to opt out at any stage. 

Since this was an exploratory study, the sample size (Table 1) was not pre-calculated, which is an accepted practice for exploratory research, as indicated in the literature [10,48]. Additionally, the sample size can be considered large enough relative to the population of Romania.

### 2.2. Study Objectives

The study used a cross-sectional, exploratory design with a correlational and descriptive approach. To achieve the main objective of understanding the relationship between socio-demographic factors and consumers’ perceptions of food promotions in Romania, the following specific objectives were formulated:

**O1:** To explore whether there are significant differences between socio-demographic factors (such as gender, age, marital status, education level, household monthly net income, and residence) and consumers’ perceptions of food promotions.
**O2:** To analyze how socio-demographic factors are related to consumers’ perceptions of food promotions.

### 2.3. Research Questions

To achieve the specific objectives of the study, a descriptive, confirmatory, and exploratory research plan was developed, leading to the formulation of the following research questions:

**RQ1.** How do specific socio-demographic factors (such as gender, age, marital status, education level, household income, and residence) differentiate consumers’ perceptions of food promotions in Romania?
**RQ2.** Are consumers’ perceptions of food promotions associated with socio-demographic predictors?

**Figure 1:**
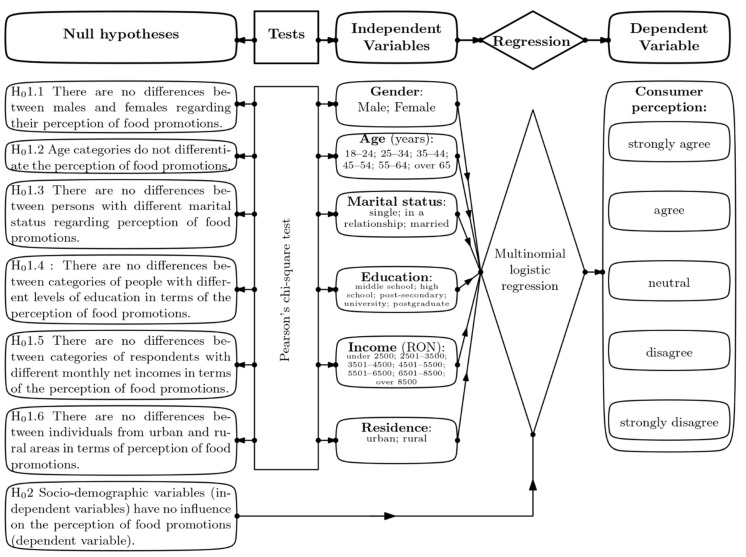
Research design flowchart.

To address these research questions, seven null hypotheses were tested, as outlined in the flowchart (Figure 1).

These research questions and hypotheses were chosen to identify which socio-demographic categories are more receptive to food promotions. Specifically, the aim was to determine which segments or target groups are more likely to agree that promotions influence their food purchasing decisions. This understanding can help tailor food promotions to better target those segments that are most responsive to them.

### 2.4. Data Analysis

The survey data were imported into R, thoroughly checked, and confirmed to be free of missing data. The statistical analysis was performed using R version 4.4.2 [49,50].

The statistical procedures used to test the hypotheses were as follows:

(i) To determine whether the observed differences among the socio-demographic categories (such as gender, age, marital status, education level, household monthly net income, and residence) in terms of the influence of promotions on purchase decisions are statistically significant, a Pearson’s chi-square test (with Cramer’s V effect size) was performed.

(ii) Regression analysis was used to examine correlations between variables. This method helps quantify the amount of change in the outcome (dependent) variable when the value of one independent variable changes while keeping the other variables constant. Regression analysis aids in understanding the relationship between variables, identifying trends and patterns in the data and making predictions for future outcomes.

The study used logistic regression for categorical outcomes, as linear regression is not suitable. Proportional odds logistic regression was ruled out due to unmet assumptions. Thus, multinomial logistic regression was chosen and implemented using the multinom() function from the *nnet* package, which employs neural network algorithms [51,52].

Both the independent and dependent variables in the multinomial logistic regression were categorical. The dependent variable consisted of five levels: *strongly disagree*, *disagree*, *neutral*, *agree*, and *strongly agree* (Figure 1).

For each predictor (independent variable), the following statistical parameters were calculated: regression coefficients (β), standard errors (S.E.), Wald z-values, associated *p*-values (Sig.), odds ratio (Exp(β)), and confidence interval (CI for Exp(β)). The model fit and goodness-of-fit were evaluated using Nagelkerke pseudo-R^2^ and the generalized Hosmer–Lemeshow test.

In both Pearson’s chi-square test and multinomial logistic regression, a *p*-value was deemed statistically significant when *p* < 0.05 [10,53].

## 3. Results

### 3.1. Descriptive Statistics

To measure the influence that promotions had on food purchasing decisions, a five-point Likert scale was used with the following distribution: strongly disagree—107 respondents (10.1%), disagree—85 respondents (8.1%), neutral—241 respondents (22.7%), agree—474 respondents (44.7%), and strongly agree—153 respondents (14.4%).

The analysis of these responses was further segmented by key socio-demographic factors, providing deeper insights into the relationships between promotions and consumer behavior, as follows:

1. *Gender*: females were more responsive to food product promotions, with a higher percentage in the *agree* (48.2% vs. 37.7%) and *strongly agree* (16.1% vs. 11.0%) categories compared to males. In contrast, males showed greater disagreement, with higher percentages in the *strongly disagree* (15.0% vs. 7.7%) and *disagree* (12.1% vs. 6.0%) categories;
2. *Age*: perception of promotions varied significantly across age groups. Young consumers (18–34 years old) were more enthusiastic about food product promotions, with high percentages in the *agree* and *strongly agree* categories. However, as age increased, respondents became more reserved, with neutral responses predominating, particularly in the 55–64 age group. Older adults (65+ years) were more critical, with higher percentages in the *strongly disagree* and *disagree* categories, highlighting generational differences in the reception of marketing strategies;
3. *Marital status*: among the respondents who selected *strongly disagree*, single individuals (10.7%) and married individuals (10.8%) were more represented compared to those in a relationship (8.9%). Conversely, at the opposite end of the scale (*strongly agree*), married individuals had the lowest share (10.2%), while individuals in a relationship (18.4%) and single individuals (15%) were more likely to *strongly agree*. Additionally, married respondents demonstrated the highest level of neutrality (27.2%), whereas those in a relationship showed the greatest enthusiasm, with 18.4% selecting *strongly agree*;
4. *Educational level*: across all educational levels, the *agree* category consistently accounted for the highest share of responses. However, participants with lower education levels showed a roughly 10% higher proportion of agreement compared to those with postgraduate education. Additionally, the percentage of respondents who selected *strongly agree* decreased as educational attainment increased, from high school education to postgraduate education.
5. *Monthly net income*: among the lower-income participants, 20.5% selected *strongly agree*, a proportion nearly double that of those who selected *strongly disagree* (10.7%). In contrast, for higher-income respondents, the trend reversed: 12.3% chose *strongly disagree*, which was nearly twice the share of those who selected *strongly agree* (6.7%).
6. *Residence*: the differences in perceptions between urban and rural respondents were minimal, with variations across all measurement levels not exceeding 3.3 percent.

### 3.2. Inferential Statistics—Hypothesis Testing

To assess whether promotions influence purchase decisions across different socio-demographic categories (gender, age, education level, net monthly income, residence, and marital status), six Pearson’s chi-square tests were conducted. Each test analyzed potential differences among socio-demographic groups based on their Likert scale responses, identifying whether promotions had a significant impact on their buying decisions.

***For RQ1: Pearson’s Chi-Square Test***

**Null** **Hypothesis** **H_0_1.1.** *There are no differences between males and females regarding their perception of food promotions*.

To test this null hypothesis, a Pearson’s chi-squared test was performed based on the contingency table (five levels of consumer perception of food promotions × two genders) presented in Table 2.

The null hypothesis H_0_1.1 was rejected based on the results. The association between gender and consumers’ perception of food promotions was statistically significant ($\chi_{\left[4\right]}^{2}=33.38$ and *p* < 0.001, Cramer’s V effect size = 0.177). The findings suggest that males exhibited a more skeptical perception of promotions, with a higher percentage in the *neutral* (24.2%, of all men) and *disagree* (12.1% of all men) categories. Conversely, females were more responsive to food product promotions, with higher percentages in the *agree* (48.1% of all women) and *strongly agree* (16.1% of all women) categories. These differences may indicate that males and females respond differently to marketing strategies, likely due to varying experiences or attitudes toward promotions. Understanding these gender-specific tendencies is critical for designing tailored promotion strategies, messaging, and approaches that cater to the preferences and behavioral patterns of each gender.

**Null** **Hypothesis** **H_0_1.2.** *Age categories do not differentiate the perception of food promotions*.

To test this null hypothesis, a Pearson’s chi-squared test was performed based on the contingency table (five consumers’ perception of food promotions × six age ranges) presented in Table 3.

The null hypothesis H_0_1.2 was rejected based on the results. The association between *age* and *consumers’ perception of food promotions* was statistically significant ($\chi_{\left[20\right]}^{2}=44.84$ and *p* = 0.001, Cramer’s V effect size = 0.103). These findings suggest that younger individuals (particularly those aged 18–24) are more likely to be influenced by food promotions. This segment exhibited the highest percentages in the *agree* and *strongly agree* categories, potentially reflecting their greater sensitivity to social influences and interest in trends. In contrast, older age groups (particularly those over 55) exhibited a more reserved perception, with higher percentages in the *neutral*, *disagree*, and *strongly disagree* categories.

These results highlight the importance of tailoring marketing strategies to different age groups. For younger consumers, promotional campaigns should focus on trends, social endorsements, and innovative approaches to capture their interest. For older age groups, more reserved and practical messaging may be more effective.

**Null** **Hypothesis** **H_0_1.3.** *There are no differences among persons with different marital status regarding perception of food promotions*.

To test this null hypothesis, a Pearson’s chi-squared test was performed based on the contingency table (five levels of consumer perception × three marital statuses) presented in Table 4.

The null hypothesis H_0_1.3 was rejected based on the analysis. The statistical analysis yielded significant results ($\chi_{\left[8\right]}^{2}=22.939$ and *p* = 0.003, Cramer’s V effect size = 0.104), indicating that marital status is associated with the perception of food promotions. Comparing the observed values with the expected ones, it was observed that only in the case of married individuals were there more respondents than expected who disagreed with the influence of promotions. In addition, the married group was the only one in which fewer respondents expressed strong agreement regarding the influence of promotions compared to the theoretical distribution. These findings suggest that married individuals exhibit a stronger reluctance to respond positively to promotions. This suggests that married individuals may be more skeptical about food promotions, potentially due to more stable purchasing habits or a different approach to marketing strategies. Understanding this trend could help tailor promotional campaigns to more effectively address the preferences and perceptions of different marital groups.

**Null** **Hypothesis** **H_0_1.4.** *There are no differences among the categories of people with different levels of education in terms of the perception of food promotions*.

To test this null hypothesis, a Pearson’s chi-squared test was performed based on the contingency table (five levels of consumer perception × five education levels) presented in Table 5.

The null hypothesis H_0_1.4 was rejected based on the results. The analysis indicated a statistically significant relationship between education level and Romanian consumers’ perception of food promotions ($\chi_{\left[16\right]}^{2}=44.38$ and *p* = 0.000, Cramer’s V effect size = 0.102). Individuals with higher education levels were found to adopt a more analytical and discerning approach, avoiding impulse purchases and focusing on fulfilling real needs. They tended to be more selective, often prioritizing a healthy, informed, and sustainable diet. The study findings indicate that individuals with a postgraduate education constituted the smallest proportion of respondents who strongly agreed that promotions influence their purchasing behavior. This suggests that higher education fosters a more critical evaluation of promotional strategies and a stronger preference for making well-informed purchasing decisions.

**Null** **Hypothesis** **H_0_1.5.** *There are no differences among the categories of respondents with different monthly net incomes in terms of the perception of food promotions*.

To test this null hypothesis, a Pearson’s chi-squared test was performed based on the contingency table (five levels of consumer perception × seven household monthly net income) presented in Table 6.

The null hypothesis H_0_1.5 was rejected based on the results. The high value of the Pearson’s chi-square test ($\chi_{\left[24\right]}^{2}=46.56$ and *p* = 0.000, Cramer’s V effect size = 0.105) indicates a statistically significant association between consumers’ perception of food promotions and household monthly net income. While consumer perception of food promotions at the highest level (*strongly agree*) varied across income categories, individuals with the highest incomes were less likely to be strongly influenced by promotions compared to other groups.

This may be due to their preference for premium, organic, or artisanal products, which are often excluded from promotional campaigns. Previous research suggests that consumers are more inclined to allocate larger budgets to high-quality products due to their superior safety and nutritional benefits [54]. Additionally, consumers with higher incomes are generally less willing to invest time in searching for promotional deals on food products, as convenience and quality often take precedence over discounted pricing.

**Null** **Hypothesis** **H_0_1.6.** *There are no differences between people in urban and rural areas in terms of the perception of food promotions*.

To test this null hypothesis, a Pearson’s chi-squared test was performed based on the contingency table (five levels of consumer perception × two residence categories) presented in Table 7.

The null hypothesis H_0_1.6 could be rejected based on the results. The Pearson’s chi-square test results ($\chi_{\left[4\right]}^{2}=3.79$ and *p* = 0.435, Cramer’s V effect size = 0.059) indicate that the observed differences in consumers’ perception of food promotions between urban and rural residents are not statistically significant. These results suggest that, despite some variations in the distribution of responses between urban and rural residents, there is no strong evidence to conclude that residence location significantly influences perceptions of food promotions. This could imply that food promotion strategies may be similarly effective in both urban and rural areas, or that other factors (such as income, education, or lifestyle) may have a greater impact on how consumers respond to promotions.

***For RQ2: Multinomial logistic regression model***

The statistical analysis was conducted using R version 4.4.2 [49,50]. The response variable, consumers’ perception of food promotions, is an ordinal outcome. Ordinal logistic regression is typically the first model choice for such data, assuming that each explanatory variable has a similar effect on the outcome [52]. This assumption, known as the proportional odds assumption, must be tested to ensure the validity of the regression model. To test this assumption, we carried out an ordinal logistic regression using the *polr()* function from the *MASS* package [51] and performed the Brant–Wald test implemented in the *brant* R package. The results of the Brant–Wald test indicate that the proportional odds assumption was not satisfied, making ordinal logistic regression not suitable for our data.

As an alternative, we employed a multinomial regression model, which is appropriate for categorical outcomes. This regression was performed using the *multinom()* function from the *nnet* package, which utilizes neural network algorithms [51,52]. The response variable consumers’ perception of food promotions was modeled as a function of all explanatory variables: *gender*, *marital status*, *education*, *age*, *residence*, and *income*. We used *strongly disagree* as the reference category. The results for the non-reference categories are presented in Table 8, Table 9, Table 10 and Table 11. All coefficients are relative to the reference category, and coefficients with suffixes such as *.L*, *.Q*, *.C*, *^4*, or higher indicate linear, quadratic, cubic, or quartic contrasts (or predictors) generated by R for ordered factor variables. For each predictor, we report the regression coefficients (β), standard errors (S.E.), Wald z-values, associated *p*-values (Sig.), odds ratio (Exp(β)), and confidence interval (CI for Exp(β)).

It can be observed from Table 8 that *gender*, *marital status*, and *education level* had a statistical impact (*p* < 0.05) on the relative odds of being in the category *disagree* vs. *strongly disagree*. It is worth mentioning that the *income* linear predictor had a *p*-value just above the significance threshold of *p* = 0.05. The base level for *marital status* is *single*; therefore, the results suggest that a shift in *marital status* toward *in a relationship* or *married* decreased the relative odds by 49%. In contrast, an increase in *income* levels doubled the relative odds of being in the “disagree” category relative to the reference category. Regarding *education*, Table 8 shows that, in addition to the linear predictor, both the quadratic and cubic predictors were also significant, indicating strong non-linear effects. The linear predictor reveals that an increase in education level significantly increased the relative odds, but there was a deceleration effect (as indicated by the quadratic predictor) followed by a positive contribution from the cubic predictor.

For the relative odds of *neutral* vs. the reference category, as depicted in Table 9, we found that *gender* significantly influenced *consumers’ perception of food promotions* (*p* = 0.012), with *females* having 54% higher relative odds of selecting the *neutral* category. The influence of education followed a similar pattern to that observed for the *disagree* vs. *strongly disagree* odds. *Income* became significant only in deceleration (*p* = 0.046) and power 6 effects (*p* = 0.015).

Table 10 shows that, all else being equal, being *male* reduced the relative odds of selecting *agree* vs. *strongly disagree* by 62%. A similar effect (63%) was observed with increasing *age;* however, the *p*-value of 0.08 was slightly above the significance threshold. Additionally, a statistically significant deceleration trend in *income* was evident (*p* = 0.018), indicating that, as *income* increased, the likelihood of selecting *agree* versus *strongly disagree* decreased at a diminishing rate.

The multinomial regression coefficients for the relative odds of *strongly agree* vs. *strongly disagree*, presented in Table 11, show statistically significant contributions from the predictors *gender* (*p* = 0.00013), *age* (*p* = 0.037), and *education level* (linear, quadratic, and cubic predictors all with *p*-values = 0). Additionally, a significant deceleration effect for the *income variable* was observed (*p* = 0.025). Based on the computed values in Table 11, we infer that being female increases the relative odds of *strongly agree* vs. the reference category by 40%. This effect is consistent with the values observed for the other relative odds, except for the *disagree* vs. *strongly disagree* odds, where the *gender* predictor was not statistically significant (*p* = 0.914). Moreover, an increase in *age* resulted in a reduction of the relative odds by 79%. The influence of *education level* mirrored the effects observed for the other relative odds except for *agree* vs. the reference category, where *education level* did not show *statistically* significant effects.

To assess the model fit, the Nagelkerke pseudo-R^2^ coefficient was calculated, resulting in a value of $R^{2}≅0.152$, indicating that the multinomial regression model explains approximately 15% of the variance in the data. For the goodness-of-fit of the model assessment, the generalized Hosmer–Lemeshow test [55], implemented in the *generalhoslem* R package [56], was performed. The results yielded $\chi^{2}$ = 25.596, *df* = 32, and a *p*-value = 0.781. A high *p*-value suggests that the model fits the data adequately.

## 4. Discussion

This study is part of a broader investigation exploring the interdependence between socio-demographic factors and various aspects of consumer behavior, such as health status, the frequency of consuming dried vegetables or products based on dried vegetables, preferences for organic versus classic chips, and consumer perceptions of food promotions. Based on the results of this study, several key findings emerge regarding the influence of socio-demographic factors on consumer behavior, particularly in relation to food promotions. These findings provide valuable insights into how variables such as gender, marital status, education level, and income affect consumer perceptions and responses to promotional offers.

*Gender:* when analyzing the hypothesis about how promotions influence males and females, the results revealed significant differences. Specifically, the percentage differences for each category showed negative differences for females (*strongly disagree*: 2.38%; *disagree*: 1.96%; *neutral*: 0.70%) and positive for males (*agree*: 6.92%; *strongly agree*: 3.46%). These results suggest that the observed values were lower than expected for females, indicating that females are more influenced by promotions than males. This finding was further supported by the multinomial logistic regression, which indicated that being female increased the relative odds of the respondents responding with *strongly agree*, *agree*, or *neutral* compared to the reference category, *strongly disagree*.

This trend can be explained by the fact that women are often more responsible for purchasing household food products than men, a phenomenon which may make them more sensitive to price changes and promotional offers. Additionally, women are more likely to engage in strategic purchasing behaviors, actively seeking sales and discounts. These behaviors could be driven by practical reasons (e.g., meeting basic survival needs such as food) and emotional factors, including satisfaction, status, and enhanced self-esteem. These motivations align with previous studies suggesting that promotions play a significant role in consumer decision-making, especially for women [6,7]. Another explanation for the results could be that women are generally more careful with their budgets, and promotions provide an opportunity to purchase more items without exceeding their financial limits. Many companies target their marketing strategies toward women, knowing that they significantly influence purchasing decisions. As a result, promotions are often designed to capture the attention of this demographic.

*Age:* the analysis of consumers’ perception of food promotions across different *age* categories revealed statistically significant differences. While opinions varied within each age group, certain trends were evident. For example, the 18–24 age group showed the largest positive percentage difference (5.16%) for the *strongly agree* response, indicating that more individuals in this age group than expected were highly influenced by promotions. In contrast, the other age categories exhibited predominantly negative percentage differences between the observed and expected values, with −1.28% for the 25–34 age group, −4.48% for the 35–44 age group, −4.72% for the 45–54 age group, and −9.76% for the 55–64 age group. An exception was observed in the over-65 age group, which showed similar positive percentage differences for *strongly disagree* (4.28%) and *agree* (5%), although this group had a smaller sample size (14 respondents).

The multinomial logistic regression analysis further supported these findings, demonstrating that being younger increased the relative odds of selecting *strongly agree* compared to the reference category. This trend can be explained through social impact and conformity theory, which suggests that younger individuals are more influenced by social interactions and trends. Young people, particularly those in the 18–24 age group, are more likely to purchase products that are popular or endorsed by peers, classmates, or colleagues. Promotions tied to social trends or interactions tend to resonate with young consumers, as they are more open to purchasing items that are perceived as trendy or socially appreciated [28,29].

Several underlying factors contribute to the higher receptiveness of young people in the 18–24 age group to promotions. Many individuals in this age group are students or early-career professionals with limited financial resources, even if they receive financial support from their parents. Promotions enable them to acquire the desired products without exceeding their budget. Additionally, social media and apps provide easy access to information about promotions, allowing users to discover discounts quickly and stay informed. Promotions are frequently shared or discussed among friends and influencers, spreading rapidly within this demographic. These promotional offers provide access to quality products, often associated with brands or social status, at a lower cost. Purchasing such products not only fulfills practical needs, but also boosts self-esteem, as many young people are conscious of how they are perceived by others.

*Marital status:* a Pearson’s chi-square test was performed to examine the influence of marital status on consumers’ perception of food promotions. The results revealed statistically significant differences between the observed and expected values. Among those who *strongly agreed* that promotions influence their perception of food products, there was a notable increase in the percentage differences: 0.57% for single respondents, 3.94% for those in a relationship, and −4.18% for married respondents.

This progression suggests that the impact of promotions on consumer perception tends to increase from single individuals to those in a relationship. However, in the married category, the percentage difference was negative, indicating that there were fewer married respondents than expected in the sample. This finding implies that married individuals are generally less influenced by promotions compared to single individuals and those in a relationship.

Additionally, results from the multinomial logistic regression analysis indicate that being in a relationship or married significantly decreased the relative odds of choosing *disagree* compared to the reference category, *strongly disagree*, by 49%. This aligns with the overall observation that marital status plays a nuanced role in shaping how consumers perceive promotional efforts.

*Education:* the analysis of the differences between observed and expected values revealed distinct variations in how individuals with different education levels perceive food promotions. Among those who strongly agreed that promotions influence their purchasing decisions, positive percentage differences were observed in the high school (7.26%), post-secondary (4.07%), and university education (0.89%) categories. However, the postgraduate category exhibited a negative difference of −5.26%. These findings suggest that respondents with lower levels of education were more likely than expected to strongly agree that promotions influence their purchasing decisions. As the education level increased, the magnitude of the positive differences diminished, indicating that individuals with higher education levels tend to be less influenced by promotions when making food product purchases.

The multinomial logistic regression analysis confirmed these findings, indicating that as the education level increased, the relative odds of agreeing about the influence of promotions on purchase decisions also increased. However, there was a strong deceleration effect as education level rose, with the quadratic predictor showing a decrease in this effect. Despite this deceleration, the cubic predictor made a positive contribution.

These trends could be attributed to the fact that individuals with higher education levels invest more time researching and purchasing food products, prioritizing informed decisions over promotional discounts. Such consumers are more likely to recognize and critically evaluate marketing strategies, making them less susceptible to promotional prices. Moreover, they often emphasize product quality, safety, and sustainability over cost savings, aligning with preferences for organic or artisanal products that are less frequently included in promotions. This aligns with existing research suggesting that, as the education level increases, consumers are more likely to prioritize long-term value and sustainability over immediate financial incentives [30,31].

*Income:* to assess whether differences between observed and expected categories (under the null hypothesis) were statistically significant, a Pearson’s chi-square test was conducted. The test value exceeded the tabular value, indicating significant differences. For individuals with lower incomes (under 2500 RON and between 2501–3500 RON), the percentage difference at the *strongly agree* level was positive. This suggests that, within the analyzed sample, more respondents than theoretically expected reported being strongly influenced by promotions. Conversely, for individuals with higher incomes (over 8000 RON), the percentage differences were negative, with these differences being larger than those observed for lower-income groups. This indicates that higher-income individuals are less likely to be influenced by promotions.

This phenomenon can be attributed to different decision-making behaviors across income levels. Lower-income individuals often rely on heuristic approaches, making quick, intuitive decisions based on “rules of thumb”, especially when under time constraints or lacking detailed information about alternatives. In such situations, promotions may be perceived as a “good deal,” offering meaningful savings to supplement households’ budget [33,34]. In contrast, higher-income individuals tend to exhibit purchasing patterns consistent with the operant conditioning theory. They are often loyal to specific brands and high-quality products, and, while they may take advantage of promotions within their preferred range of products, they are not dependent on discounts due to their financial flexibility [35,36].

The multinomial logistic regression analysis showed that income had a statistically significant (*p* < 0.05) deceleration effect on the relative odds of selecting *strongly agree*, *agree*, or *neutral* versus the reference category, *strongly disagree*. This finding suggests that, as the quadratic income predictor increases, individuals’ susceptibility to food promotions decreases when making purchasing decisions.

*Residence:* to assess whether urban and rural residents differ significantly in their perception of food promotions, a Pearson’s chi-square test was performed. The analysis revealed no statistically significant differences, indicating that both groups share similar perceptions. This similarity may be attributed to factors such as equal access to food products, shared sources of information, and similar motivations to save money. Additionally, the growing prevalence of online shopping and home delivery services has made promotions and delivery options equally attractive in both urban and rural areas. This shift was further supported by the multinomial logistic regression, which confirmed that residence had no statistically significant effect on food promotion perception, indicating that consumers in both settings are similarly influenced by these promotional offers.

The findings of this study provide valuable insights into consumer behavior, particularly into how factors like gender, age, education, and income shape perceptions of food promotions. Understanding these factors enables businesses to tailor their marketing strategies to specific consumer segments more effectively. For instance, identifying which groups are more responsive to promotions can help businesses design more targeted and impactful promotional campaigns.

Additionally, understanding the motivations behind food purchase decisions, such as the role of price and perceived value, allows businesses to refine their overall approach. This knowledge extends beyond marketing to enhance broader operational processes, including product returns and recovery procedures, ultimately improving customer satisfaction and driving profitability [57]. By recognizing how consumers perceive food quality based on price [58,59], companies can better align their product offerings and customer service strategies.

Whether for large corporations or small businesses, effective marketing and customer-centered strategies are crucial to success [60,61,62]. Studies suggest that consumers often associate small traders with a stronger focus on maintaining a good reputation, which underscores the need for trust-building practices in the marketplace. This is especially relevant when considering how promotional strategies and consumer perceptions influence both consumer loyalty and the trust placed in businesses, regardless of their size [63]. Understanding these dynamics is essential for fostering long-term customer relationships and improving business outcomes.

## 5. Conclusions

This study was designed with the premise that a deep understanding of consumer behavior, particularly in relation to socio-demographic factors, is crucial for shaping effective marketing strategies and enhancing consumer satisfaction. To capture the nuances of consumer perception regarding food promotions, a five-point Likert scale (strongly disagree, disagree, neutral, agree, strongly agree) was employed.

Pearson’s chi-square tests were conducted to identify whether various socio-demographic factors could serve as predictors of consumer perception of food promotions. The results revealed significant differences in consumer responses based on gender, age, education level, marital status, and monthly net income. However, residence was the only socio-demographic factor that did not demonstrate a statistically significant impact on food promotion perception.

The multinomial logistic regression analysis further corroborated these findings, showing that all socio-demographic factors, except for residence, had a statistically significant influence on consumers’ perception of food promotions. Gender and marital status were identified as strong predictors, with females and single individuals more likely to be influenced by promotions. Education level showed a fluctuating trend, with individuals holding higher education being more responsive to promotions. However, this effect was tempered by deceleration effects. Similarly, income exhibited a deceleration effect, with individuals less likely to be influenced by promotions as the quadratic income predictor increased. The study highlights the importance of understanding consumer motivations and socio-demographic factors when designing marketing strategies. By identifying the key predictors of consumer perception of food promotions, companies can better anticipate shifts in consumer behavior and develop targeted campaigns that align with the unique needs and preferences of different demographic groups. While promotions can be effective for certain segments, their impact varies depending on how each group perceives the value, quality, and the significance of discounts. For this reason, marketers must consider a complex combination of demographic factors, including gender, marital status, income, and education level when designing promotional campaigns for food products.

One limitation of this study is the exclusive use of an online questionnaire, which may have excluded individuals without internet access or those unfamiliar with technology, potentially skewing the sample. Additionally, online surveys can lead to inattentive or inaccurate responses, affecting data reliability. These factors should be considered when interpreting the results. To improve accuracy in future research, a broader range of demographic, psychographic, and geographic samples is needed, as different samples may yield varying results. Multiple studies will be necessary to validate and strengthen the findings. Successful marketing strategies require an understanding of consumers’ life stages and motivations. Marketers must tailor messaging to meet the specific needs and perceptions of their target audiences, with personalization and strategic integration of promotional elements being crucial to effective campaigns.

## Figures and Tables

**Table 1:** The respondents’ socio-demographic characteristics.

Characteristics	Share in the Sample	N	Percentage (%)
Gender	Female	713	67.3
	Male	347	32.7
Age (years)	18–24	434	40.9
	25–34	235	22.1
	35–44	192	18.1
	45–54	144	13.6
	55–64	41	3.9
	Over 65	14	1.4
Marital status	Single	300	28.3
	In a relationship	370	34.9
	Married	390	36.8
Education level	Middle school education	2	0.2
	High school education	106	10.0
	Post-secondary education	27	2.5
	University education	653	61.6
	Postgraduate education	272	25.7
Household monthly net income (RON)	Under 2500	205	19.3
	2501–3500	188	17.7
	3501–4500	177	16.8
	4501–5500	104	9.8
	5501–6500	95	9.0
	6501–8500	104	9.8
	Over 8500	187	17.6
Residence	Urban	777	73.3
	Rural	283	26.7

**Table 2:** Consumers’ perception of food promotions in relation to gender.

			Consumers’ Perception of Food Promotions					Total Lines
			Strongly Disagree	Disagree	Neutral	Agree	Strongly Agree	
Gender	Male	Count	52	42	84	131	38	347
		Expected count	35.0	28	79	155	50	347
		% within a *	15.0	12.1	24.2	37.7	11.0	100
		% within b *	48.6	49.4	34.9	27.6	24.8	32.7
	Female	Count	55	43	157	343	115	713
		Expected count	72.0	57	162	319	103	713
		% within a	7.7	6.0	22.0	48.2	16.1	100
		% within b	51.4	50.6	65.1	72.4	75.2	67.3
	Total columns	Count	107	85	241	474	153	1060
		Expected count	107	85	241	474	153	1060
		% within c *	10.1	8.1	22.7	44.7	14.4	100
		% within b	100	100	100	100	100	100

* a—gender (number of respondents corresponding to the respective gender); b—consumers’ perception of food promotions (number of respondents corresponding to the respective level of perception of promotions); c—total respondents (regardless of gender).

**Table 3:** Consumers’ perception of food promotions in relation to age.

			Consumers’ Perception of Food Promotions					Total Lines
			Strongly Disagree	Disagree	Neutral	Agree	Strongly Agree	
Age	18–24	Count	37	39	71	202	85	434
		Expected count	43.8	34.8	98.7	194.1	62.6	434
		% within a *	8.5	9	16.4	46.5	19.6	100
		% within b *	34.6	45.9	29.5	42.6	55.6	40.9
	25–34	Count Expected count % within a % within b	25	16	64	99	31	235
			23.7	18.8	53.4	105.1	34	235
			10.6	6.8	27.3	42.1	13.2	100
			23.4	18.8	26.6	20.9	20.3	22.2
	35–44	Count	17	18	48	90	19	192
		Expected count	19.5	15.4	43.7	85.8	27.6	192
		% within a	8.8	9.4	25	46.9	9.9	100
		% within b	15.9	21.2	19.9	19	12.4	18.1
	45–54	Count	17	9	41	63	14	144
		Expected count	14.5	11.6	32.7	64.4	20.8	144
		% within a	11.8	6.2	28.5	43.7	9.7	100
		% within b	15.9	10.6	17	13.3	9.1	13.6
	55–64	Count	9	2	15	13	2	41
		Expected count	4.1	3.3	9.3	18.3	6	41
		% within a	21.9	4.9	36.6	31.7	4.9	100
		% within b	8.4	2.3	6.2	2.7	1.3	3.9
	Over 65	Count	2	1	2	7	2	14
		Expected count	1.4	1.1	3.2	6.3	2	14
		% within a	14.3	7.1	14.3	50	14.3	100
		% within b	1.8	1.2	0.8	1.5	1.3	1.3
	Total columns	Count	107	85	241	474	153	1060
		Expected count	107	85	241	474	153	1060
		% within c *	10.1	8.1	22.7	44.7	14.4	100
		% within b	100	100	100	100	100	100

* a—age (number of respondents corresponding to the respective age); b—consumers’ perception of food promotions (number of respondents corresponding to the respective level of perception of promotions); c—total respondents (regardless of age).

**Table 4:** Consumers’ perception of food promotions in relation to marital status.

			Consumers’ Perception of Food Promotions					Total Lines
			Strongly Disagree	Disagree	Neutral	Agree	Strongly Agree	
Marital status	Single	Count	32	34	61	128	45	300
		Expected count	30.3	24.1	68.2	134.1	43.3	300
		% within a *	10.7	11.3	20.3	42.7	15	100
		% within b *	29.9	40.0	25.3	27.0	29.4	28.3
	In a relationship	Count Expected count % within a % within b	33	30	74	165	68	370
			37.3	29.7	84.1	165.5	53.4	370
			8.9	8.1	20.0	44.6	18.4	100
			30.8	35.3	30.7	34.8	44.5	34.9
	Married	Count	42	21	106	181	40	390
		Expected count	39.4	31.2	88.7	174.4	56.3	390
		% within a	10.8	5.4	27.2	46.4	10.3	100
		% within b	39.3	24.7	44.0	38.2	26.1	36.8
	Total columns	Count	107	85	241	474	153	1060
		Expected count	107	85	241	474	153	1060
		% within c *	10.1	8.1	22.7	44.7	14.4	100
		% within b	100	100	100	100	100	100

* a—marital status (number of respondents corresponding to the respective marital status); b—consumers’ perception of food promotions (number of respondents corresponding to the respective level of perception of promotions); c—total respondents (regardless of marital status).

**Table 5:** Consumers’ perception of food promotions in relation to education level.

			Consumers’ Perception of Food Promotions					Total Lines
			Strongly Disagree	Disagree	Neutral	Agree	Strongly Agree	
Education level	Middle school level	Count	1	0	0	1	0	2
		Expected count	0.2	0.2	0.5	0.9	0.3	2.1
		% within a *	50	0	0	50	0	100
		% within b *	0.9	0	0	0.2	0	0.2
	High school education	Count Expected count % within a % within b	10	5	15	53	23	106
			10.7	8.5	24.1	47.4	15.3	106
			9.4	4.7	14.2	50	21.7	100
			9.3	5.9	6.2	11.2	15	10
	Post-high-school education	Count	1	4	6	11	5	27
		Expected count	2.7	2.1	6	11.8	3.8	26.4
		% within a	3.7	14.8	22.2	40.8	18.5	100
		% within b	0.9	4.7	2.5	2.3	3.3	2.5
	University education	Count	67	59	127	300	100	653
		Expected count	65.9	52.4	148.5	292	94.2	653
		% within a	10.3	9.1	19.4	45.9	15.3	100
		% within b	62.7	69.4	52.7	63.3	65.4	61.6
	Postgraduate education	Count	28	17	93	109	25	272
		Expected count	27.4	21.8	61.8	121.7	39.3	272
		% within a	10.3	6.2	34.2	40.1	9.2	100
		% within b	26.2	20	38.6	23	16.3	25.7
	Total columns	Count	107	85	241	474	153	1060
		Expected count	107	85	241	474	153	1060
		% within c *	10.1	8.1	22.7	44.7	14.4	100
		% within b	100	100	100	100	100	100

* a—education level (number of respondents corresponding to the respective educational level); b—consumers’ perception of food promotions (number of respondents corresponding to the respective level of perception of promotions); c—total respondents (regardless of the level of education).

**Table 6:** Consumers’ perception of food promotions in relation to household monthly net income.

			Consumers’ Perception of Food Promotions					Total Lines
			Strongly Disagree	Disagree	Neutral	Agree	Strongly Agree	
Household monthly net income (RON)	Under 2500	Count	22	14	31	96	42	205
		Expected count	20.7	16.4	46.6	91.7	29.6	205
		% within a *	10.7	6.8	15.1	46.9	20.5	100
		% within b *	20.6	16.5	12.9	20.3	27.5	19.3
	2501–3500	Count Expected count % within a % within b	24	12	40	75	37	188
			19.0	15.1	42.7	84.1	27.1	188
			12.7	6.4	21.3	39.9	19.7	100
			22.4	14.1	16.6	15.8	24.2	17.8
	3501–4500	Count	8	15	42	90	22	177
		Expected count	17.9	14.2	40.2	79.1	25.6	177
		% within a	4.5	8.5	23.7	50.9	12.4	100
		% within b	7.5	17.6	17.4	19.0	14.4	16.7
	4501–5500	Count	11	9	22	45	17	104
		Expected count	10.5	8.4	23.6	46.5	15.0	104
		% within a	10.6	8.7	21.1	43.3	16.3	100
		% within b	10.3	10.6	9.1	9.5	11.1	9.8
	5501–6500	Count	7	6	28	45	9	95
		Expected count	9.6	7.6	21.6	42.5	13.7	95
		% within a	7.3	6.3	29.5	47.4	9.5	100
		% within b	6.5	7.1	11.6	9.5	5.9	8.9
	6501–8500	Count	12	11	21	47	13	104
		Expected count	10.5	8.4	23.6	46.5	15.0	104
		% within a	11.5	10.6	20.2	45.2	12.5	100
		% within b	11.2	12.9	8.7	9.9	8.5	9.8
	Over 8500	Count	23	18	57	76	13	187
		Expected count	18.9	15.0	42.5	83.6	27.0	187
		% within a	12.3	9.6	30.5	40.6	7.0	100
		% within b	21.5	21.2	23.6	16.0	8.5	17.6
	Total columns	Count	107	85	241	474	153	1060
		Expected count	107	85	241	474	153	1060
		% within c *	10.1	8.1	22.7	44.7	14.4	100
		% within b	100	100	100	100	100	100

* a—household monthly net income (number of respondents corresponding to the respective income); b—consumers’ perception of food promotions (number of respondents corresponding to the respective level of perception of promotions); c—total respondents (regardless of income).

**Table 7:** Consumers’ perception of food promotions in relation to residence.

			Consumers’ Perception of Food Promotions					Total Lines
			Strongly Disagree	Disagree	Neutral	Agree	Strongly Agree	
Residence	Urban	Count	81	57	177	343	119	777
		Expected count	78.4	62.3	176.7	347.4	112.2	777
		% within a *	10.4	7.3	22.8	44.2	15.3	100
		% within b *	75.7	67.1	73.4	72.4	77.8	73.3
	Rural	Count Expected count % within a % within b	26	28	64	131	34	283
			28.6	22.7	64.3	126.6	40.8	283
			9.2	9.9	22.6	46.3	12.0	100
			24.3	32.9	26.6	27.6	22.2	26.7
	Total columns	Count	107	85	241	474	153	1060
		Expected count	107	85	241	474	153	1060
		% within c *	10.1	8.1	22.7	44.7	14.4	100
		% within b	100	100	100	100	100	100

* a—residence (number of respondents corresponding to the respective residence); b—consumers’ perception of food promotions (number of respondents corresponding to the respective level of perception of promotions); c—total respondents (regardless of residence).

**Table 8:** Regression coefficients and corresponding statistics for the category *disagree* relative to the reference category.

	B	S.E.	Wald z	Sig.	Exp(B)	95% C.I. for EXP(B)	
						Lower	Upper
**Gender**							
* Male*	−0.033	0.305	−0.109	0.914	0.967	0.533	1.757
**Age**							
* Age.L*	−0.739	0.88	−0.839	0.401	0.478	0.085	2.681
* Age.Q*	−0.080	0.794	−0.102	0.919	0.922	0.194	4.377
* Age.C*	0.371	0.688	0.539	0.59	1.449	0.376	5.578
* Age^4*	0.519	0.59	0.88	0.3788	1.68	0.529	5.338
* Age^5*	−0.283	0.444	−0.636	0.525	0.754	0.315	1.801
**Marital Status**							
* Marital status.L*	−0.665	0.329	−2.021	0.043	0.514	0.27	0.98
* Marital status.Q*	−0.17	0.284	−0.599	0.549	0.844	0.484	1.472
**Education Level**							
* Education level.L*	7.040	0.347	20.276	0.0	1142.154	578.3075	2255.747
* Education level.Q*	−7.225	0.57	−12.674	0.0	7.284 × 10^−4^	2.383 × 10^−4^	2.226 × 10^−3^
* Education level.C*	3.174	0.363	8.739	0.0	23.893	11.726	48.682
* Education level^4*	0.444	0.922	0.4815	0.630	1.559	0.256	9.497
**Income**							
* Income.L*	0.766	0.406	1.885	0.059	2.151	0.97	4.77
* Income.Q*	−0.634	0.408	−1.554	0.12	0.531	0.239	1.18
* Income.C*	0.168	0.423	0.399	0.69	1.183	0.517	2.709
* Income^4*	0.149	0.407	0.366	0.714	1.16	0.523	2.575
* Income^5*	−0.594	0.468	−1.27	0.204	0.552	0.22	1.382
* Income^6*	0.509	0.479	−0.636	0.287	1.664	0.651	4.253
**Residence**							
* Urban*	−0.427	0.341	−1.249	0.211	0.653	0.3342	1.275

**Table 9:** Regression coefficients and corresponding statistics for the category *neutral* relative to the reference category.

	B	S.E.	Wald z	Sig.	Exp(B)	95% C.I. for EXP(B)	
						Lower	Upper
**Gender**							
* Male*	−0.619	0.246	−2.519	0.012	0.539	0.333	0.872
**Age**							
* Age.L*	−0.849	0.694	−1.223	0.221	0.428	0.11	1.668
* Age.Q*	−0.195	0.636	−0.307	0.759	0.823	0.236	2.862
* Age.C*	0.047	0.492	0.097	0.923	1.049	0.4	2.749
* Age^4*	−0.074	0.384	−0.192	0.848	0.929	0.438	1.972
* Age^5*	−0.03	0.312	−0.096	0.923	0.9704	0.527	1.788
**Marital Status**							
* Marital status.L*	0.118	0.257	0.458	0.647	1.125	0.68	1.86
* Marital status.Q*	0.06	0.231	0.261	0.794	1.062	0.6759	1.669
**Education Level**							
* Education level.L*	9.749	0.271	35.972	0.0	17,137.45	10,075.31	29,149.7
* Education level.Q*	8.345	0.521	−16.026	0.0	2.376 × 10^−4^	8.563 × 10^−5^	6.593 × 10^−4^
* Education level.C*	4.776	0.278	17.188	0.0	118.575	68.785	204.406
* Education level^4*	−0.584	0.861	−0.678	0.498	0.558	0.103	3.014
**Income**							
* Income.L*	0.246	0.334	0.735	0.462	1.278	0.664	2.46
* Income.Q*	−0.661	0.332	−1.993	0.046	0.516	0.27	0.989
* Income.C*	0.344	0.341	1.008	0.313	1.41	0.723	2.75
* Income^4*	0.278	0.33	0.841	0.4	1.32	0.691	2.521
* Income^5*	−0.024	0.379	−0.063	0.95	0.976	0.465	2.052
* Income^6*	0.945	0.3889	2.431	0.015	2.573	1.2	5.515
**Residence**							
* Urban*	−0.275	0.285	−0.966	0.334	0.759	0.435	1.327

**Table 10:** Regression coefficients and corresponding statistics for *agree* relative to the reference category.

	B	S.E.	Wald z	Sig.	Exp(B)	95% C.I. for EXP(B)	
						Lower	Upper
**Gender**							
* Male*	−0.967	0.229	−4.22	2.441 × 10^−5^	0.38	0.243	0.596
**Age**							
* Age.L*	−0.99	0.584	−1.694	0.09	0.372	0.118	1.168
* Age.Q*	0.46	0.534	0.862	0.389	1.584	0.556	4.514
* Age.C*	0.621	0.437	1.421	0.155	1.86	0.79	4.377
* Age^4*	0.599	0.366	1.635	0.102	1.82	0.888	3.729
* Age^5*	0.098	0.299	0.326	0.744	1.103	0.613	1.983
**Marital Status**							
* Marital status.L*	0.247	0.241	1.025	0.306	1.28	0.798	2.051
* Marital status.Q*	0.112	0.214	0.523	0.601	1.118	0.736	1.7
**Education Level**							
* Education level.L*	1.118	0.954	1.171	0.242	3.057	0.471	19.841
* Education level.Q*	−1.681	0.992	−1.694	0.09	0.186	0.027	1.302
* Education level.C*	0.807	0.531	1.518	0.129	2.241	0.791	6.352
* Education level^4*	0.424	0.824	0.514	0.607	1.528	0.304	7.69
**Income**							
* Income.L*	0.063	0.305	0.207	0.836	1.065	0.586	1.937
* Income.Q*	−0.729	0.308	−2.366	0.018	0.482	0.264	0.882
* Income.C*	0.165	0.318	0.517	0.605	1.179	0.632	2.2
* Income^4*	0.249	0.301	0.827	0.408	1.282	0.711	2.312
* Income^5*	−0.371	0.359	−1.033	0.301	0.69	0.341	1.395
* Income^6*	0.808	0.366	2.207	0.027	2.245	1.095	4.601
**Residence**							
* Urban*	−0.118	0.265	−0.447	0.655	0.888	0.529	1.493

**Table 11:** Regression coefficients and corresponding statistics for *strongly agree* relative to the reference category.

	B	S.E.	Wald z	Sig.	Exp(B)	95% C.I. for EXP(B)	
						Lower	Upper
**Gender**							
* Male*	−1.081	0.283	−3.814	1.366 × 10^−4^	0.339	0.195	0.591
**Age**							
* Age.L*	−1.565	0.751	−2.084	0.037	0.209	0.048	0.911
* Age.Q*	0.677	0.666	1.017	0.309	1.968	0.534	7.2580
* Age.C*	0.74	0.618	1.198	0.23	2.095	0.625	7.031
* Age^4*	0.737	0.558	1.32	0.186	2.09	0.7	6.243
* Age^5*	0.302	0.421	0.718	0.473	1.353	0.593	3.083
**Marital Status**							
* Marital status.L*	0.195	0.293	0.667	0.505	1.216	0.684	2.16
* Marital status.Q*	−0.066	0.25	−0.263	0.793	0.936	0.574	1.528
**Education Level**							
* Education level.L*	8.238	0.284	28.999	0.0	3781.358	2166.946	6598.535
* Education level.Q*	−8.28	0.543	−15.241	0.0	2.534 × 10^−4^	8.737 × 10^−5^	7.35 × 10^−4^
* Education level.C*	4.633	0.274	16.935	0.0	102.834	60.153	175.797
* Education level^4*	−0.696	0.877	−0.793	0.428	0.498	0.089	2.782
**Income**							
* Income.L*	−0.521	0.372	−1.399	0.162	0.594	0.286	1.232
* Income.Q*	−0.844	0.376	−2.245	0.025	0.43	0.206	0.898
* Income.C*	0.18	0.386	0.465	0.641	1.197	0.561	2.553
* Income^4*	0.011	0.359	0.032	0.975	1.012	0.501	2.043
* Income^5*	−0.388	0.427	−0.908	0.364	0.678	0.294	1.568
* Income^6*	0.278	0.431	0.645	0.519	1.32	0.567	3.073
**Residence**							
* Urban*	0.323	0.316	1.022	0.307	1.382	0.743	2.569

## Data Availability

The data presented in this study are available on request from the corresponding author. The data are not publicly available due to privacy restrictions.
